# Determination of the Optimal Wavelength of the Hemolysis Index Measurement

**DOI:** 10.3390/jcm12185864

**Published:** 2023-09-09

**Authors:** Akiyo Ishiguro, Mitsuaki Nishioka, Akihiro Morishige, Mai Yoneshiro, Kanae Shinkawa, Aki Fujinaga, Toshihiko Kobayashi, Yutaka Suehiro, Takahiro Yamasaki

**Affiliations:** 1 Department of Oncology and Laboratory Medicine, Yamaguchi University Graduate School of Medicine, Ube 755-8505, Japan; ishi-a@yamaguchi-u.ac.jp (A.I.); ysuehiro@yamaguchi-u.ac.jp (Y.S.); 2 Division of Laboratory, Yamaguchi University Hospital, Ube 755-8505, Japan; mnishi@yamaguchi-u.ac.jp (M.N.); amorishi@yamaguchi-u.ac.jp (A.M.); y-m1129@yamaguchi-u.ac.jp (M.Y.); kanae-s@yamaguchi-u.ac.jp (K.S.); akimasa@yamaguchi-u.ac.jp (A.F.); kobatosi@yamaguchi-u.ac.jp (T.K.)

**Keywords:** hemolysis index, optimal wavelength, bilirubin, chyle

## Abstract

Many biochemical auto-analyzers have methods that measure the hemolysis index (HI) to quantitatively assess the degree of hemolysis. Past reports on HI are mostly in vitro studies. Therefore, we evaluated the optimal wavelength of HI measurement ex vivo using clinical samples. Four different wavelengths (410/451 nm: HI-1, 451/478 nm: HI-2, 545/596 nm: HI-3 and 571/596 nm: HI-4) were selected for HI measurement, and correlations were examined from the measurement results of 3890 clinical samples. Another set of 9446 clinical samples was used to examine the correlation of HI with lactate dehydrogenase (LDH), aspartate aminotransferase (AST) and potassium (K). Strong correlations were found between HI-4 and HI-1 and between HI-4 and HI-3. HI-1 and HI-2 cannot correctly assess hemolysis for high bilirubin samples, and HI-3 cannot correctly assess hemolysis for high triglyceride samples. LDH, AST and K correlated positively with HI-4 in clinical samples. For every 1-unit increase in HI-4, LDH increased by 19.51 U/L, AST by 1.03 U/L and K by 0.061 mmol/L, comparable to reports of other studies. In clinical samples, HI-4 was less susceptible to bilirubin and chyle and reflected well the changes in LDH, AST and K caused by hemolysis. This suggested that the optimal wavelength for HI measurement is 571 nm.

## 1. Introduction

Hemolysis is defined as the membrane disruption of red blood cells due to various physical, chemical and biological factors with the subsequent release of intracellular components such as hemoglobin to outside the cells [1]. There are three possible factors by which in vitro hemolysis (or iatrogenic hemolysis) can cause errors in biochemical test values: (1) the release of erythrocyte constituents resulting in some increased values in serum, (2) dilution by the release of erythrocyte constituents resulting in decreased values in serum and (3) hemoglobin interference itself, e.g., by affecting the colorimetric quantitation of the constituents [2]. With respect to the three biochemical test items that are most susceptible to hemolysis (lactate dehydrogenase: LDH, aspartate aminotransferase: AST and potassium: K), the ratios of the concentrations inside and outside the erythrocyte are large. It is reported that they are 160 times larger for LDH, 40 times larger for AST and 22.7 times larger for K inside than outside the erythrocyte [3,4,5]. Hemolysis may cause errors in biochemical test results, which can often have an adverse impact on data reliability. In particular, K is a component that is greatly associated with the support of life, and unless pseudohyperkalemia caused by hemolysis can be identified, treatment for hyperkalemia may be conducted that can actually cause hypokalemia and may invite confusion in healthcare settings [6]. The determination of hemolyzed samples used to be evaluated visually, but visual detection is ambiguous, is subject to individual variation and, hence, is unreliable [7,8]. In addition, the visual detection of hemolysis is difficult if turbidity or an elevated bilirubin concentration is present in the samples.

In recent years, many biochemical auto-analyzers have included a built-in feature to quantitatively and objectively measure the hemolysis index (HI), which evaluates the degree of hemolysis of the serum, thus making it possible to detect hemolysis more objectively [9]. When an examiner encounters abnormal values in a biochemical test, it is important to know whether it is a pathological change. If abnormal values due to errors in pre-analytical processes such as blood collection techniques or sample transportation are reported, clinicians may make incorrect decisions. Approximately 60% of the abnormal values caused by errors in the pre-analytical process are due to hemolysis [10]. Therefore, the HI is very useful in these determinations. In recent years, the automation of specimen pre-processing has developed rapidly. In large clinical laboratories, the process of loading the specimen into the specimen transport system, sample centrifugation and analysis is fully automated. Conventionally, the process of centrifugation was performed by an examiner, and the confirmation of serum status including hemolysis could be visually checked before analysis. However, in the situation of a fully automated system, the examiner cannot visually check the serum status before analysis due to full automation of the pre-analytical processes. Therefore, the importance of the HI measurement is increasing even more.

Some analyzers, such as the Roche Cobas c501 and Siemens Dimension Vista 1500, have reported reference ranges of HI for each device [9], but no such reference ranges have been established for other analyzers. Also, the wavelength used for measuring HI differs by device and laboratory [9]. Currently, HI is not standardized, and thus, there are no clear standards for detecting hemolysis. The need for the standardization of clinical laboratory test items is widely known, and they are being standardized internationally. However, unlike other laboratory test items, HI is not being standardized. The measurement wavelength, reagent composition, calculation method, reporting unit and judgment method of HI measurement are not standardized. Furthermore, there is no common cut-off value or guideline for HI. To utilize the HI reliably, it is necessary to consider the determination of HI and to set the cut-off value of HI at each facility. However, these considerations and settings are very complicated. Therefore, the effective utilization of HI is not widespread. If the wavelengths used in HI measurements can be standardized and a clear and evidence-based evaluation method can be established for hemolyzed samples, HI can be used more effectively in clinical settings.

Regarding the impact of hemolysis on clinical laboratory data, there are reports of in vitro studies conducted using artificially hemolyzed samples [11,12,13,14,15]. The reported methods to artificially prepare hemolyzed samples include using combination freeze/thaw cycles and osmotic pressure difference [11,12] and the physical breakdown of red blood cells [13,14,15]. In our previous study [16], we devised a new method to artificially prepare hemolyzed samples by physically breaking down red blood cells using stainless steel beads and a tissue crusher. From the results of studying the effect of hemolysis using hemolyzed samples prepared artificially with this method, we reported a wavelength of 571 nm to be the most appropriate [16]. Previous reports on hemolysis studies used artificially hemolyzed samples and did not mention validation using clinical samples [11,12,13,14,15,16]. However, the degree of impact of hemolysis may differ for samples hemolyzed artificially in vitro and clinical samples, and it is thus necessary to confirm whether the method can also be applied to clinical samples. Therefore, in the present study, we used clinical samples to determine whether our reported wavelength of 571 nm for HI measurement is optimal and whether this HI measurement adequately reflects the impact of hemolysis in clinical samples.

## 2. Materials and Methods

### 2.1. Measuring Instruments and Reagents

Measurement of the HI was performed using the JCA-BM6070, an automatic analyzer from the JCA-BM series (JEOL Ltd., Akishima, Tokyo, Japan: hereinafter, BM6070), by referring to the literature [16]. The reagent for HI measurement comprised 500 μL of Cuvette Conditioner ECO (JEOL Ltd., Akishima, Tokyo, Japan) containing a surfactant mixed with 500 mL of saline. The BM6070 is designed to measure biochemical test items after diluting the serum 5 times with saline. As with other biochemical test items, HI is also measured after dilution 5 times with saline. After mixing 7.5 μL of diluted serum with 60 μL of HI measurement reagent and measuring the absorbance of the mixture, HI was calculated using the following formula.

HI = 440.54 × (Absorbance (B) − 0.8967 × Absorbance (A))

Absorbance (A) at 658/694 nm (main wavelength/sub-wavelength).

Absorbance (B) at 410/451 nm, 451/478 nm, 545/596 nm or 571/596 nm (main wavelength/sub-wavelength).

**Figure 1:**
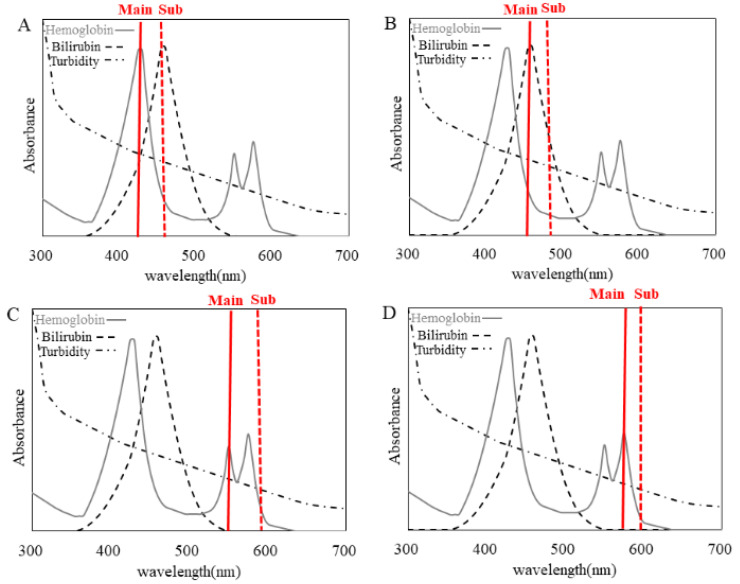
Absorbance spectrum of hemoglobin, bilirubin and turbidity. The absorbance spectrum of hemoglobin is indicated by the gray line, bilirubin by the dashed line and turbidity spectrum by the dash-dotted line. The measured wavelengths (main/sub) are shown for (**A**) HI-1 (410/451 nm), (**B**) HI-2 (451/478 nm), (**C**) HI-3 (545/596 nm) and (**D**) HI-4 (571/596 nm).

Each Absorbance (A) and Absorbance (B) is obtained by subtracting the absorbance of the sub-wavelength from that of the main wavelength. In biochemical analyzers, two-wavelength analysis is performed by setting a main wavelength and a sub-wavelength to avoid the effects of coexisting substances such as bilirubin or turbidity, as well as dirt and scratches on the cuvette. Absorbance (A) was used to reduce the effect of turbidity. To determine the optimal wavelength for HI measurement that can avoid the effects of bilirubin, turbidity, etc., we decided to conduct a comparative experiment by setting four different wavelengths. For Absorbance (B), four measurement wavelengths (410/451 nm: HI-1, 451/478 nm: HI-2, 545/596 nm: HI-3 and 571/596 nm: HI-4) were considered (Figure 1). The measurement items and measurement reagents are shown in Table 1. K was analyzed using an EA08M automated electrolyte analyzer (A&T Co., Ltd., Yokohama, Kanagawa, Japan), and the other items were analyzed with the BM6070.

### 2.2. HI Measurements of Clinical Samples at the Four Different Wavelengths

Four different wavelengths were set when the HI of the 3890 samples was measured at our hospital between 5 June and 14 June 2019. The main wavelength used for HI-1 was 410 nm, at which hemoglobin has its highest absorption peak; that for HI-2 was 451 nm, at which bilirubin has its highest absorption peak; that for HI-3 was 545 nm, at which hemoglobin has its second absorption peak, and that for HI-4 was 571 nm, at which hemoglobin has its third absorption peak (Figure 1). Because HI-4 was the optimal wavelength according to our previous report for which artificially hemolyzed samples were used [16], we examined the correlation between HI-4 and HI-1 to HI-3 using HI-4 as a reference. These patient samples were drawn for clinical purposes and were to be discarded. This study was approved by the Ethics Committee of Yamaguchi University Hospital (H2019-068).

### 2.3. Correlation of LDH, AST and K with HI in Clinical Samples

To properly evaluate only the changes in AST, LDH and K due to hemolysis, not the changes in AST, LDH and K due to diseases with pathological abnormalities, reference ranges for biochemical items were established, and the effect of HI changes due to hemolysis on AST, LDH and K was evaluated in samples that met the reference ranges. Of the 127,241 samples measured at our hospital during an 11-month period from September 2015 to July 2016, 9446 samples that met the requirements were used to investigate the correlation between LDH, AST and K with HI-4. This study was approved by the Ethics Committee of Yamaguchi University Hospital (H28-082).

### 2.4. Statistical Analysis

Data analyses were performed using StatFlex v6 (Artech Co., Ltd., Osaka City, Osaka, Japan).

## 3. Results

### 3.1. HI Measurement of Clinical Samples at Different Wavelengths

**Figure 2:**
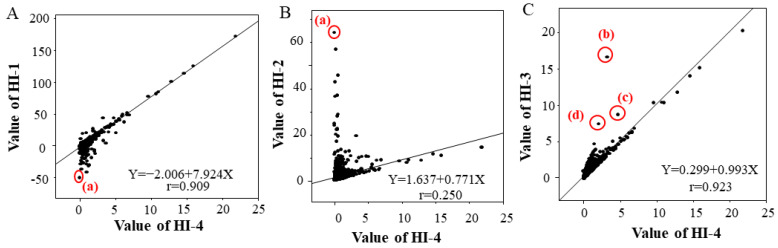
Correlation of HI-1 with HI-4 (**A**), HI-2 with HI-4 (**B**), and HI-3 with HI-4 (**C**) in the clinical data. The linear regression equation and the single correlation coefficient are shown within each graph. Biochemical test values of the four samples (a–d) circled in red that deviated significantly from the correlations are shown in Table 2.

The single correlation coefficients (r) between HI-4 and HI-1, HI-2 and HI-3 were 0.909 for HI-1, 0.250 for HI-2 and 0.923 for HI-3. There was a strong correlation between HI-4 and HI-1 and between HI-4 and HI-3, but no correlation was found between HI-4 and HI-2 (Figure 2). Sample (a) in Figure 2A,B has a high T-BIL value (Table 2). The HI-1 values of sample (a) showed lower deviation compared to the correlation chart of HI-4 and HI-1, and the HI-2 values of sample (a) showed higher deviation compared to the correlation chart of HI-4 and HI-2. Samples (b), (c) and (d) in Figure 2C have high triglyceride levels and high turbidity (Table 2). The HI-3 values of samples (b), (c) and (d) showed higher deviation compared to the correlation chart of HI-4 due to chyle (Figure 2C, Table 2).

### 3.2. Correlation of LDH, AST and K with HI in Clinical Samples

**Figure 3:**
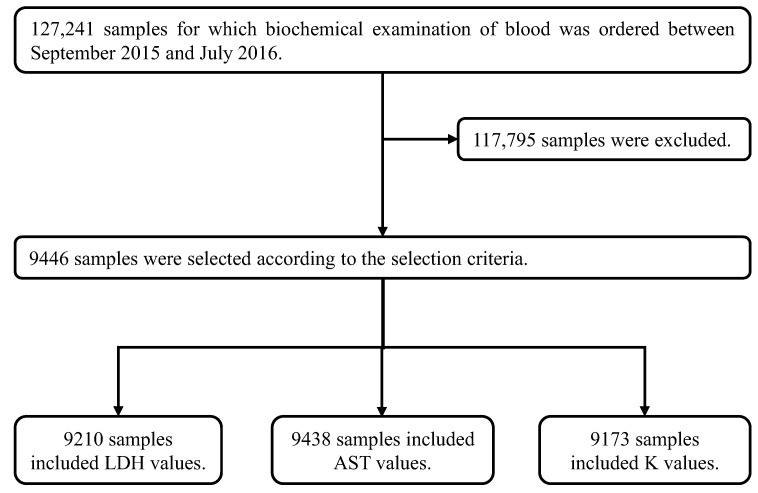
Method for extracting clinical data. The 127,241 clinical samples were collected from patients for whom biochemical examinations of blood were ordered. After their collection, 117,795 samples were excluded because at least one or more items in these samples were out of the ranges shown in Table 3 (the selection criteria). Items in the remaining 9446 selected samples were completely within the ranges shown in Table 3. In these 9446 samples, the number of samples with data for LDH was 9210, for AST was 9438 and for K was 9173.

**Figure 4:**
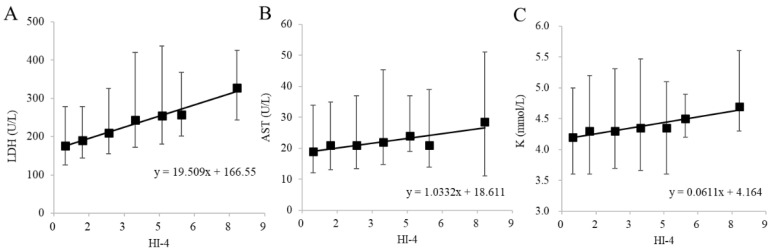
Relationship of biochemical items of (**A**) LDH, (**B**) AST and (**C**) K with HI-4 in the clinical data. The black squares represent median values of patient clinical data for each HI group. The upper and lower bars indicate the 25th and 75th percentile values. The linear regression equation is shown within each graph.

The results of biochemical tests performed on 127,241 samples at our hospital during an 11-month period from September 2015 to July 2016 were collected. After collection, 117,795 samples that deviated from the selection criteria range for any one of the test items listed in Table 3 were excluded. The selection criteria range in Table 3 was established based on the Common Reference Range proposed by the JCCLS (Japanese Committee for Clinical Laboratory Standards) and was modified appropriately [17]. Proper evaluation of hemolysis is not possible if patient data with elevated values of LDH, AST or K due to diseases with pathological abnormalities such as hemolytic anemia, renal disease and liver disease are used. Hence, the selection criteria range in Table 3 was established to exclude such samples. We tried, as much as possible, to ensure that variations in LDH, AST and K due only to hemolysis were analyzed. Among the samples, 9446 met all of the requirements of the selection criteria range in Table 3. The relationship of HI-4 with LDH, AST and K, which are the items most susceptible to hemolysis, was investigated. Of the 9446 samples that met the requirements, 9210 samples could be used for LDH, 9438 samples for AST and 9173 samples for K (Figure 3). These samples were classified into seven groups (HI-4 < 1, 1 ≤ HI-4 < 2, 2 ≤ HI-4 < 3, 3 ≤ HI-4 < 4, 4 ≤ HI-4 < 5, 5 ≤ HI-4 < 6 and 6 ≤ HI-4) based on HI-4 values, referring to the method of Mansour et al. [18]. The median increase in LDH, AST and K for each group was calculated by non-parametric methods. Every one-unit increase in HI-4 corresponded to a 19.51 U/L increase in LDH, a 1.03 U/L increase in AST and a 0.061 mmol/L increase in K (Figure 4). We compared the in vitro increase in K, AST and LDH per unit increase in HI-1 in our previous report [16] and the ex vivo increase in the clinical samples of the present study. When comparing the previous report with the current results, the change values of LDH (U/L), AST (U/L) and K (mmol/L) were +14.42/+19.51, +0.87/+1.03 and +0.058/+0.061, respectively (previous/current). These results suggested that the previous and current values were comparable.

## 4. Discussion

According to our previous report [16], in which four different wavelengths for HI measurement were set and artificially hemolyzed samples were used, a value of around 571 nm was considered to be the optimal wavelength. Mansour et al. examined two models of the effect of hemolysis in clinical samples, assuming that artificially hemolyzed samples that non-selectively destroy all cells cannot explain the trend in clinical specimens [18]. However, they did not mention the optimal wavelength for HI measurement, and they also included patient samples containing pathological changes in clinical samples; thus, it is possible that samples containing extreme pathological changes in K, LD and AST may not correctly assess changes due to hemolysis. Therefore, we conducted this study to confirm the validity of the optimal wavelength for HI measurement and to evaluate the effect of hemolysis in clinical samples without obvious pathological changes rather than in artificially hemolyzed samples.

In the present study, we compared HI measured at four wavelengths to determine whether 571 nm (HI-4) is also the optimal wavelength for clinical samples. Regarding the correlation between HI-4 and HI-1 and between HI-2 and HI-3, strong correlations were observed between HI-4 and HI-1 and between HI-4 and HI-3. It was suggested that the absorption peaks of hemoglobin should be adopted as the main wavelength in HI measurement. In the measurement at HI-1, the main wavelength was set to the first absorption peak of hemoglobin, but the sub-wavelength overlaps with the absorption peak of bilirubin (Figure 1A). HI-1 in sample (a) shows significant negative bias compared to HI-4 in sample (a) due to a high T-BIL value (Figure 2A, Table 2). Therefore, it is assumed that HI-1 is susceptible to bilirubin. In Figure 2B, HI-2 and HI-4 values in sample (a) have deviated significantly. This is thought to result from the main wavelength of HI-2 overlapping with the maximum absorption peak of bilirubin, due to which HI-2 is considered to show significant positive bias compared to HI-4 in sample (a). Therefore, it is assumed that HI-2 is also susceptible to bilirubin. In Figure 2C, three samples, (b), (c) and (d) with chyle, showed higher deviation in HI-3 than in HI-4. In the absorption spectrum of chyle, the difference in absorbance of the main wavelength and that of the sub-wavelength is larger for HI-3 than for the other HIs (Figure 1). Thus, the impact of chyle on HI-3 is thought to be larger. In fact, in the three samples (b), (c) and (d), HI-3 values showed a positive deviation because of the large absorbance of turbidity due to hyperlipidemia (Table 2). These results indicate that HI-1, HI-2 and HI-3 affect HI measurements in samples with hyperbilirubinemia and samples with chyle. In contrast, HI-4 was found to be less susceptible to bilirubin and chyle, making it a suitable condition for HI measurement. In our previous report [16], we evaluated the influence of commercially available interference components on the four wavelengths used for HI measurement and found that HI-1 and HI-2 were influenced by free and conjugated bilirubin; HI-1, HI-2 and HI-3 were influenced by chyle and HI-4 showed no influence of these interference components, which is consistent with our findings using clinical samples.

We previously reported an increase in K, AST and LDH per unit increase in HI-1 in in vitro experiments using artificially hemolyzed samples [16]. In the present study, we analyzed the increase in K, AST and LDH per unit increase in HI-1 ex vivo using approximately 10,000 clinical samples. Comparison of the increase in K, AST and LDH per unit increase in HI-1 in vitro and ex vivo showed similar changes. Other researchers have reported that the amount of change in K per 100 mg/dL of Hb is 0.3–0.5 mmol/L [11,19]. In our previous report [16], we presented the correlation equation (ΔHb (mg/dL) = ΔHI-4 × 14.76 + 0.80), where Δ indicates the amount of change. Using this correlation equation and the correlation equation in Figure 4C, we calculated the amount of change in K per 100 mg/dL of Hb in the clinical samples of the present study to be 0.41 mmol/L, which is consistent with the reports by others; hence, we considered the results of this study using clinical samples to be valid.

There is no consensus among instrument suppliers on such issues as how to detect interference components (hemoglobin) in the measurement of HI, how to select wavelengths, how to calculate HI and how to set reference values, and thus, caution is required when using HI calculated by different instruments [20]. Although acceptable ranges of HI have been reported for some instruments [9], the measurement wavelengths and HI calculation formulae are different for each instrument, and there is no common range for all instruments. Although for some instruments, a change of 10% or more from the baseline value is considered a significant difference as per the criteria for HI, it has been reported that a common reference value for all items overestimates hemolysis [12,20]. Regarding the selection of a wavelength for HI measurement, our present study and previous report suggest that the optimal wavelength for HI measurement is around 571 nm [16]. However, a limitation of the present study is that only one type of instrument was used. Further studies with several other instruments are needed to verify the reported results of the present study.

The harmonization of HI is necessary for the effective utilization of HI and to set appropriate cutoff levels. The recommendations of the Working Group for Preanalytical Phase (WG-PRE) of the European Federation of Clinical Chemistry and Laboratory Medicine (EFLM) propose a practical approach to managing clinical chemistry test results for hemolyzed samples [21]. These recommendations state that to improve HI harmonization, device-specific units indicating the degree of hemolysis should be converted to g/dL (cell-free hemoglobin). It also recommended that HI-specific controls be used to monitor HI on an ongoing basis [21]. Although there is a limitation in the availability of HI-specific controls, once these quality control substances become widely available in the diagnostic drugs market, it would be desirable to monitor the analytical performance of HI. Continuous reporting of appropriately controlled HI values may be useful in monitoring patients with hemolytic anemia and other pathological conditions, although further studies are needed to distinguish between in vivo hemolysis (or pathological hemolysis) and in vitro hemolysis (or iatrogenic hemolysis). In addition, as hemolysis is a suitable quality indicator for collected blood samples [22], HI may also be useful as an indicator for the evaluation and improvement of pre-analytical processes. With further harmonization of HI in regard to wavelength, reporting unit (e.g., g/dL), reference values and control samples for HI measurement, it will become possible to use HI more effectively and appropriately in various instances, such as quality control in clinical and laboratory settings.

## 5. Conclusions

On the basis of the results of this study using clinical samples, we suggest that the optimal wavelength for HI measurement is 571 nm.

## Figures and Tables

**Table 1:** Measurement items and principal methods of measurement.

Abbreviation *^1^	Analyte Name	Unit	Method	Product Name	Reagent Manufacturer
LDH	Lactate dehydrogenase	U/L	Enzymatic method *^2^	L-type LD.J	FUJIFILM Wako Pure Chemical Co., Ltd. Osaka City, Osaka, Japan
AST	Aspartate aminotransferase	U/L	Enzymatic method *^2^	L-type AST.J2	FUJIFILM Wako Pure Chemical Co., Ltd. Osaka City, Osaka, Japan
ALT	Alanine aminotransferase	U/L	Enzymatic method *^2^	L-type ALT.J2	FUJIFILM Wako Pure Chemical Co., Ltd. Osaka City, Osaka, Japan
GGT	γ-glutamyltransferase	U/L	Enzymatic method *^2^	Quick Auto Neo γ-GT JS	Shino-Test Corp. Chiyoda, Tokyo, Japan
TP	Total proteins	g/dL	Biuret method	Aqua-auto Kainos TP-II	Kainos Laboratories, Inc. Bunkyo, Tokyo, Japan
ALB	Albumin	g/dL	Advanced BCP method	Aqua-auto Kainos ALB	Kainos Laboratories, Inc. Bunkyo, Tokyo, Japan
TG	Triglyceride	mg/dL	FG eliminating enzyme method	Determiner L TG II	Minaris Medical Co., Ltd. Chuo, Tokyo, Japan
TC	Total cholesterol	mg/dL	Cholesterol oxidase method	Determiner L TC II	Minaris Medical Co., Ltd. Chuo, Tokyo, Japan
HDL-C	HDL cholesterol	mg/dL	Selective inhibition method	MetaboLead HDL-C	Minaris Medical Co., Ltd. Chuo, Tokyo, Japan
LDL-C	LDL cholesterol	mg/dL	Selective solubilization method	MetaboLead LDL-C	Minaris Medical Co., Ltd. Chuo, Tokyo, Japan
UN	Urea nitrogen	mg/dL	Urease GLDH method	Aqua-auto Kainos UN-II	Kainos Laboratories, Inc. Bunkyo, Tokyo, Japan
UA	Uric acid	mg/dL	Uricase–POD method	Determiner L UA	Minaris Medical Co., Ltd. Chuo, Tokyo, Japan
CRE	Creatinine	mg/dL	Enzymatic method	Cygnus Auto CRE	Shino-Test Corp. Chiyoda, Tokyo, Japan
T-BIL	Total bilirubin	mg/dL	Vanadate oxidation method	Total Bilirubin E HA	FUJIFILM Wako Pure Chemical Co., Ltd. Osaka City, Osaka, Japan
K	Potassium	mmol/L	Ion-selective electrode method	EA08M *^3^	A & T Co., Ltd. Yokohama City, Kanagawa, Japan

*^1^: The abbreviation are indicated abbreviations of the analyte name. *^2^: JSCC-recommended method. *^3^: Name of the automated electrolyte analyzer.

**Table 2:** Biological test values of the four circled samples (a-d) in Figure 2.

Sample No.	(a)	(b)	(c)	(d)
Picture of serum	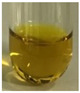	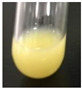	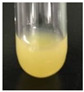	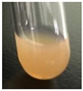
TG (mg/dL)	167.6	684.8	480.6	1230.9
TC (mg/dL)	141.7	100.9	224.4	210.3
HDL-C (mg/dL)	8.7	44.6	25.4	21.6
LDL-C (mg/dL)	31.2	22.8	138.9	5.9
T-BIL (mg/dL)	28.1	1.2	0.4	0.67

The biochemical data of the 4 samples with discrepancies in the 4 types of HI values are indicated. Data in red indicate abnormally high values.

**Table 3:** Selection criteria for clinical data.

Item	Male	Female
Age (years)	18–70	
TP (g/dL)	6.4–8.1	
ALB (g/dL)	3.7–5.1	
CRE (mg/dL)	0.60–1.15	0.40–0.85
UA (mg/dL)	3.5–8.2	2.5–6.0
ALT (U/L)	10–45	7–27
GGT (U/L)	13–70	9–36
UN (mg/dL)	7–24	

Except for age, the ranges for these items refer to the ranges published by the JCCLS [17].

## Data Availability

All data relevant to the study are included in this article. Further inquiries can be directed to the corresponding author (T.Y.).
